# Screening of 22q11.2DS Using Multiplex Ligation-Dependent Probe Amplification as an Alternative Diagnostic Method

**DOI:** 10.1155/2020/6945730

**Published:** 2020-09-28

**Authors:** Sathiya Maran, Siti Aisyah Faten, Swee-Hua Erin Lim, Kok-Song Lai, Wan Pauzi Wan Ibrahim, Ravindran Ankathil, Siew Hua Gan, Huay Lin Tan

**Affiliations:** ^1^Human Genome Centre, School of Medical Sciences, Universiti Sains Malaysia, 16150 Kubang Kerian, Kelantan, Malaysia; ^2^School of Pharmacy, Monash University, Jalan Lagoon Selatan 47500 Bandar Sunway Selangor Darul Ehsan, Malaysia; ^3^Health Sciences Division, Abu Dhabi Women's College, Higher Colleges of Technology, 41012 Abu Dhabi, UAE; ^4^Department of Paediatrics, School of Medical Sciences, Universiti Sains Malaysia, 16150 Kubang Kerian, Kelantan, Malaysia; ^5^Faculty of Medicine and Health Sciences, Universiti Sultan Zainal Abidin, 20400 Kuala Terengganu, Terengganu, Malaysia

## Abstract

**Background:**

The 22q11.2 deletion syndrome (22q11.2DS) is the most common form of deletion disorder in humans. Low copy repeats flanking the 22q11.2 region confers a substrate for nonallelic homologous recombination (NAHR) events leading to rearrangements which have been reported to be associated with highly variable and expansive phenotypes. The 22q11.2DS is reported as the most common genetic cause of congenital heart defects (CHDs).

**Methods:**

A total of 42 patients with congenital heart defects, as confirmed by echocardiography, were recruited. Genetic molecular analysis using a fluorescence *in situ* hybridization (FISH) technique was conducted as part of routine 22q11.2DS screening, followed by multiplex ligation-dependent probe amplification (MLPA), which serves as a confirmatory test.

**Results:**

Two of the 42 CHD cases (4.76%) indicated the presence of 22q11.2DS, and interestingly, both cases have conotruncal heart defects. In terms of concordance of techniques used, MLPA is superior since it can detect deletions within the 22q11.2 locus and outside of the typically deleted region (TDR) as well as duplications.

**Conclusion:**

The incidence of 22q11.2DS among patients with CHD in the east coast of Malaysia is 0.047. MLPA is a scalable and affordable alternative molecular diagnostic method in the screening of 22q11.2DS and can be routinely applied for the diagnosis of deletion syndromes.

## 1. Introduction

The 22q11.2 deletion syndrome (22q11.2DS) is the most common genetic disorder caused by deletions of chromosome 22, at the q11.2 locus [1]. Depending on the method of accreting in different countries, prevalence of 22q11.2DS has been reported to range from 1 : 2000 to 1 : 7000 [2].

Approximately 97% of patients with 22q11.2DS were reported to harbour the 3 Mb deletion of DNA, causing a haploinsufficiency in about 30-40 genes within the locus [3, 4]. Common clinical features of 22q11.2DS include dysmorphic facies, congenital heart defects, palatal malformations, learning difficulties, and immunodeficiency. In terms of congenital heart defects (CHDs), the 22q11.2DS has been reported as a common genetic cause, contributing to approximately 1.9% of CHDs at birth [4]. About 70% of CHDs are conotruncal malformations, followed by tetralogy of Fallot (20%), truncus arteriosus (6%), and conoventricular ventricular septal defect (VSD) (14%), which is a type B interruption of the aortic arch (IAA) (13%) [5, 6]. Nevertheless, incidences in atrial septal defects (ASDs), pulmonary valve stenosis (PVS), hypoplastic left heart syndrome (HLHS), double-outlet right ventricle, and transposition of the great arteries (TGA) are less common [4].

The 22q11.2 deletion syndrome presents an expansive phenotype with more than 180 clinical features involving almost every organ and system in the body [2]. Thus, diagnoses through clinical features are unreliable, leading to a heavy reliance on molecular genetic analysis where chromosome 22q11.2 is observed for deletions and/or duplications using a cytogenetic approach with the method regarded as highly reliable [7].

Fluorescence *in situ* hybridization (FISH), a molecular genetic analysis which detects chromosomes for abnormalities, has been reported as the “gold standard” for diagnosis [8]. FISH utilises fluorescence probes (N25 and *TUPLE1*) located at the proximal part of the typically deleted region (TDR) to determine abnormalities within the 22q11.2 regions. However, FISH probes cannot detect deletions proximal or distal to the particular probe used; besides, it only provides information on targeted locations [9, 10]. Therefore, it does not allow a comprehensive evaluation of the whole genome. In addition, it is a challenge to identify atypical smaller deletions by FISH due to the fact that the probes are unable to cover these regions. Hence, FISH alone cannot provide reliable diagnosis for cases of 22q11.2DS, thus necessitating the need for an alternative molecular genetic diagnostic tool which could provide a scalable and accurate diagnosis in a cost-effective and less labour-intensive manner with practicality for application in small laboratories.

Over the years, new diagnostic methods for the detection of the 22q11.2 deletion syndrome have been developed, including comparative genomic hybridization (CGH) [11, 12], multiplex ligation-dependent probe amplification (MLPA) [13], multiplex quantitative real-time polymerase chain reaction (PCR) [14], and high-resolution single-nucleotide polymorphism (SNP) microarray analysis [14, 15]. Nonetheless, some of these methods are still at the experimental stage, requiring expensive equipment for assay and data analysis as well as trained personnel to conduct the experiments. On the other hand, the MLPA technique can easily be performed in laboratories without such needs. MLPA is a PCR-based technique which can provide a good resolution combined with practicality and affordability, thus providing approximately 98.9% sensitivity and 97.8% specificity [16].

Both FISH and MLPA techniques are locus-specific tests. However, FISH is a qualitative test that indicates the presence or absence of the 22q11.2DS. On the other hand, MLPA provides both qualitative and copy number variation data for the 22q11.2 region and other locus contained in the kit.

Despite the significance and high prevalence of 22q11.2DS as one of the most common frequent genomic disorders [17], to the best of our knowledge, the incidence of 22q11.2DS in Malaysia has not been reported. Therefore, a pilot approach is necessary in determining the incidence of 22q11.2DS in the east coast of Malaysia and in investigating the utility of MLPA as a potential alternative to FISH in diagnosing 22q11.2DS among nonsyndromic patients with CHDs.

## 2. Material and Methods

### 2.1. Editorial Policies and Ethical Considerations

The research project was approved by the Research and Ethics Committee, School of Medical Sciences, Universiti Sains Malaysia (USM) Health Campus (USMKK/PPP/JEPeM [252.3(13)]), and the Ministry of Health Malaysia (KKM/NIHSEC/BOO-2/2/2/P13-147) which complies with the Declaration of Helsinki. Written informed consent was obtained from either the parents of patients below 18 years old or directly from the patients who are 18 years old and above. Additionally, all patients/parents of patients must sign a written informed consent form to allow publication of their medical and/or genetic information.

### 2.2. Study Population and Sample Collection

CHD patients admitted to Hospital Universiti Sains Malaysia (HUSM), which serves as the main tertiary cardiac referral centre in the east coast region of Peninsular Malaysia from January 2013 to November 2014, were recruited (*n* = 42). Patients ranging from newborns to adults confirmed to harbour the defect based on an echocardiogram were recruited. The conditions were identified and confirmed by a paediatric cardiologist in the Echocardiography Unit, HUSM.

Approximately 3 ml of peripheral blood was collected from each patient; 1 ml of the sample was stored in a sodium-heparin tube for culture of lymphocytes, whereas 2 ml was stored in EDTA tubes for DNA extraction.

#### 2.2.1. FISH

FISH analysis of chromosome 22q11.2 was performed on metaphase spreads and on interphase nuclei obtained from the synchronous culture of lymphocytes, using a commercially available DiGeorge/VCFC *TUPLE1* probe (Cytocell, Cambridge, UK). The DiGeorge/VCFC *TUPLE1* region deletion probe measures approximately 120 kb of the gene and covers the entire *TUPLE1* gene as well as the flanking DNA. The 22qter sub-telomere-specific probe (clone N85A3) is located in the *ProSAP2/SHANK3* gene, allowing identification of the most distal 22q13.3 deletions. In a normal cell, there should be two red and two green signals (2R and 2G, respectively), while a deletion of the DGCR probe target will result in only the formation of 1R and 2G signals. On the other hand, a deletion of the 22q subtelomeric probe will result in 2R and 1G signals. The slide preparation, denaturation, and hybridization were carried out according to the manufacturer's protocols (http://www.amplitech.net/PDF/microdeletions/LPU004.pdf). Generally, 20 metaphases were examined and 100 interphase nuclei were scored for the number of signals present.

#### 2.2.2. MLPA

Genomic DNA was extracted from peripheral blood using the GeneAll® Exgene™ Blood SV mini kit (GeneAll, Korea) following the manufacturer's instructions. DNA concentration and purity were determined using the NanoQuant spectrophotometer (Tecan, USA). MLPA was conducted using the SALSA MLPA P250-A1 DiGeorge Kit (MRC Holland, Amsterdam, Netherlands). The kit consisted of 48 probes from which 29 are within the 22q11.2 loci while the remaining 19 are within the regions of DiGeorge syndrome (DGS) and DGS type II (all covering chromosomes 22q13, 4q, 8p, 9q, 10p, and 17p).

PCR amplification was carried out at the Human Genome Centre, Universiti Sains Malaysia, Kelantan, Malaysia. The capillary electrophoresis using an ABI Prism 3100 Genetic Analyzer (Applied Biosystems, Foster City, CA) was conducted at First BASE Laboratories Sdn Bhd, Malaysia. The data were analysed using the Coffalyser VBA analysis software V8 (http://www.mlpa.com/coffalyser). Two healthy controls (as confirmed by echocardiography) were used as positive samples for data normalisation. The threshold for deletion was set at 0.75 while the threshold for duplication was set at 1.30. Samples which showed deletions and/or duplications were reanalysed for further confirmation.

## 3. Results

**Figure 1:**
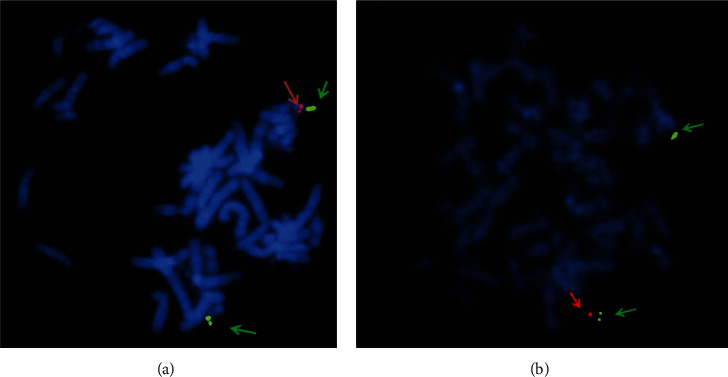
Photographs of FISH assays showing microdeletions of chromosome 22q11.2 in (a) S1 and (b) S2. A metaphase spread indicating the presence of two green signals designating *SHANK3* (indicated by a green arrow) and a single red signal designating the 22q11.2 region (indicated by a red arrow).

To our knowledge, this study is the first to successfully report screening of 42 nonsyndromic CHD patients for deletions and/or duplications in the 22q11.2 locus using both the FISH assay and MLPA tests, as a part of the routine diagnosis of 22q11.2DS in Malaysia. The screening was followed by a MLPA test, which was performed on all patients irrespective of their FISH assay results, thus serving as a confirmatory test. From the 42 cases, two samples (4.76%) showed deletion and duplications within the 22q11.2 regions. Subsequently, the FISH assay using the DiGeorge/VCFC *TUPLE1* probe (Cytocell, Cambridge, UK) in both patients showing deletions within the 22q11.2 regions was reconducted (Figure 1) for further confirmation, whereas 40 cases showed no deletion and/or duplications using both FISH and MLPA techniques.

The 22q11.2 deletions were detected in two patients using the DiGeorge/VCFC *TUPLE1* probe (Cytocell, Cambridge, UK) (Figure 1). The MLPA assay using the SALSA MLPA P250-A1 DiGeorge Kit (MRC Holland, Amsterdam, Netherlands) also confirmed deletion in both patients. However, in contrast to FISH, MLPA detected duplications within the 22q11.2 region indicating that it is a more sensitive tool for detection of duplications.

**Figure 2:**
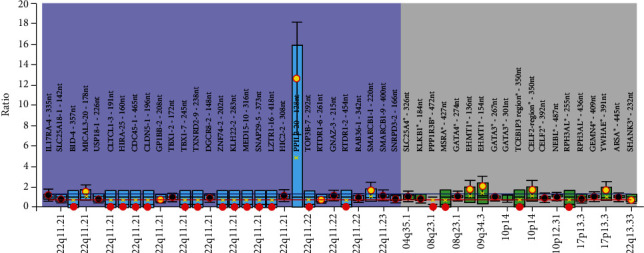
An MLPA ratio chart showing deletions of *LZTR1* (LCR C-D), *TOP3B* (LCR D-E), *RTDR1* (LCR D-E), *BID4*, *PPP1R3B*, *MSRA*, *TCEB1P3*, and *RPH3AL* as well as duplications of *PPIL2*, *EHMT1*, and *YWHAE*. The black dots display the probe ratios and the error bars with 95% confidence intervals. The red dots display deletion, and the yellow dots display duplication. The blue box plots display genes within the 22q11.2 region whereas the green box plots display other regions associated with 22q11.2DS. A map view of all the locations are displayed on the *x*-axis while the *y*-axis shows the ratio. The red and blue horizontal lines indicate the arbitrary borders for loss and gains at a ratio of 0.7 and 1.3, respectively.

When the patients' data was further analysed, the first patient (S1) was a 3-week-old baby girl diagnosed with patent ductus arteriosus. The MLPA assay showed deletions in the typically deleted regions (TDR): *LZTR1* (LCR C-D) and *TOP3B* (LCR D-E) and *RTDR1* (LCR D-E). In addition, deletions detected by the probes outside of 22q11.2 regions were also observed: *BID4* (22q11.2 CES), *PPP1R3B* and *MSRA* located within the 8p23.1 locus, *TCEB1P3* (chromosome 10p14), and *RPH3AL* (chromosome 17p13.3) alongside duplication of *EHMT1* (chromosome 9q34.3), *CELF2* (chromosome 10p14), and *YWHAE* (chromosome 17p13.3) (Figure 2).

**Figure 3:**
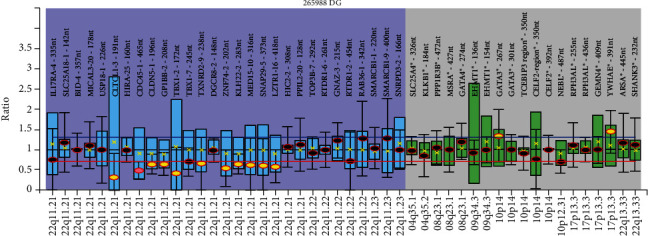
An MLPA ratio chart showing a 3 Mb deletion from *CLTCL1* to *LZTR1* and duplication of *YWHAE*. The black dots display the probe ratios and the error bars with 95% confidence intervals. The blue box plots display genes within the 22q11.2 region whereas the green box plots display other regions associated with 22q11.2DS. The red and blue horizontal lines indicate the arbitrary borders for loss and gains at a ratio of 0.7 and 1.3, respectively. The yellow dots below the red horizontal line indicate duplication of the gene whereas the yellow dots above the blue horizontal lines indicate duplication of the gene. Map view locations are displayed on the *x*-axis while the *y*-axis shows the ratio results.

The second patient (S2) was a month-old baby boy diagnosed with pulmonary atresia with VSD and a major aortopulmonary collateral artery (MAPCAS). The MLPA assay detected a 3 Mb deletion within the TDR from *CLTCL1* (LCR A-B) to *LZTR1* (LCR C-D) as well as duplication of *YWHAE* (chromosome 17p13.3) and *GATA3* (chromosome 10p14) (Figure 3).

## 4. Discussion

In this study, we successfully screened the 22q11.2DS in 42 nonsyndromic Malaysian CHD patients using both the FISH and MLPA techniques. MLPA confirmed the presence of deletions as detected by the FISH assay in the two nonsyndromic CHD patients. Nevertheless, in both cases, FISH failed to detect deletions located outside the TDR and deletions in probes outside of the 22q11.2 regions as well as duplications indicating that MLPA is superior to FISH as a diagnostic tool. Our findings suggest the possibility of using MLPA as a potential alternative diagnostic method in the screening of 22q11.2DS.

S1 carried a deletion within the distal deletion region and deletions outside of the 22q11.2 TDR. Since the “classical” candidate genes were not deleted, the cardiac malformation observed might be due to the deletion of the other genes. Furthermore, deletions were also observed in *BID4*, a gene associated with the cat eye syndrome, as well as genes outside of chromosome 22q11.2: *PPP1R3B*, *MSRA*, *TCEB1P3*, and *RPH3AL*. This is defined as a typical characteristic of molecular complexity which controls the 22q11.2DS phenotypes. Furthermore, the 22q11.2 hemizygosity alone cannot explain the genetic mechanism of the highly variable phenotypic expression of 22q11.2DS. McDonald-McGinn and colleagues [18] proposed that the mechanism of 22q11.2DS involves a combined effect of multigene deletion and a stochastic phenomenon which includes the sensitivity of individual genes within the 22q11.2 region to gene dosage [19, 20], variants in genes on the intact 22q11.2 [1], and additional “modifying” variants outside the 22q11.2 region [8]. S2 carried a 3 Mb deletion from LCR A and LCR D, and approximately 90% of patients with 22q11.2DS have been reported to harbour this deletion [21]. The result is also in agreement with Carotti and colleagues [22] who reported that up to 40% of patients with MAPCAs have DiGeorge syndrome with chromosome 22q11.2 deletion indicating that the deletion contributes to the occurrence of the disease.

It is noteworthy that in both of the patients, duplication of *YWHAE* was observed. The FISH assay failed to detect this duplication; this is due to the fact that the DiGeorge/VCFC *TUPLE1* probe (Cytocell, Cambridge, UK) used in this study does not contain a probe for *YWHAE* [13]. Studies have reported that individuals with duplications within *YWHAE* were characterised by a mild neurocognitive and pervasive developmental disorder phenotype in the presence of minor craniofacial abnormalities [23, 24]. This is in agreement with previous reports that individuals with 22q11.2DS have high rates of cognitive and psychiatric problems [25, 26]. In terms of craniofacial abnormalities, only mild but typical facial, skeletal, and dental characteristics, including significant retrusion of the lower part of the face, were observed [27]. However, due to unavailability of phenotypic characteristics of patients in this study, the cognitive and psychiatric characteristics could not be further assessed.

A large proportion (95%) of the recruited subjects in our study did not show deletions and/or duplications, within the 22q11.2DS, despite the use of both MLPA and FISH assays. This occurrence might be explained by the fact that the patients may carry very small deletions or even point mutations, which are below the resolution of the methods used. Alternatively, the patients may have other microdeletion or microduplication syndromes.

In the present study, all recruited patients were examined by two different approaches, enabling first-hand experience in comparing the techniques and the underlying principles of each technique, hence leading to a conclusion that MLPA stands superior to FISH based on the mentioned criteria. Our conclusion is also at par with the findings from Jalali and colleagues [13] who reported that in the near future, MLPA will be able to replace 22q FISH.

The MLPA assay compared to FISH is relatively simple to be used in clinical laboratories of small- or medium-scale dimension with much cheaper reagent cost per assay [28]. A recent report by Sorensen and colleagues [29] even suggested that the MLPA technique is used within paediatric cardiology as a first-tier screen in detecting clinically relevant copy number variants (CNV) and in identifying syndromic patients at an early stage. Another advantage of MLPA is that it does not require cell culturing, which is a tedious technique and often requires trained professional for aseptic handlings, with high contamination issues. The use of DNA as a starting material in MLPA provides additional benefit to a certain group of patients who are reluctant or are unable to provide blood samples, where saliva and hair root samples can be used as alternatives. Furthermore, MLPA can potentially diagnose a broader spectrum of abnormalities [30].

Although the FISH technique is still in routine use in many laboratories, it cannot detect deletions that are either proximal or distal to the particular probe used [21]. Another major downfall of FISH is that both the interphase and metaphase FISH can only detect known genetic aberrations, provided that the specific probe is available [31]. Moreover, owing to limitations in resolution, FISH analysis has been reported to be unable to detect microdeletions or microduplications smaller than 40 kb [32]. Our small data may add to the body of evidence of current findings in determining alternative diagnostic methods for 22q11.2DS screening. A larger cohort to further confirm the concordance of these techniques and provide the prevalence rate of 22q11.2DS in Malaysia is suggested in the future.

## 5. Conclusions

The incidence of 22q11.2DS in the east coast of Malaysia is estimated as 0.047 with the samples collected in this study. Our study highlighted the scalability, concurrency, and applicability of MLPA as a potential alternative to the FISH assay in detecting 22q11.2DS. Compared to FISH, the MLPA method can be conducted with ease, is less time-consuming, and is less laborious.

## Data Availability

The cytogenetic and MLPA data used to support the findings of this study are available from the corresponding author upon request.
